# VP1 of Enterovirus 71 Protects Mice Against Enterovirus 71 and Coxsackievirus B3 in Lethal Challenge Experiment

**DOI:** 10.3389/fimmu.2019.02564

**Published:** 2019-11-08

**Authors:** Fang-Hong Chen, Xiong Liu, Hua-Li Fang, Nan Nan, Zhan Li, Nian-Zhi Ning, De-Yan Luo, Tao Li, Hui Wang

**Affiliations:** ^1^State Key Laboratory of Pathogens and Biosecurity, Beijing Institute of Microbiology and Epidemiology, Beijing, China; ^2^Department of Microbiology, Anhui Medical University, Anhui, China; ^3^PLA Center for Disease Control and Prevention, Beijing, China

**Keywords:** HFMD, Enterovirus 71, Coxsackievirus B3, cross-protection, VP1 protein

## Abstract

Enterovirus and Coxsackievirus are the major viruses that cause hand, foot, and mouth disease (HFMD) outbreaks worldwide. Several studies have shown the potential of viral envelope protein 1 (VP1) on providing protective effects from viral strains of different genotypes. However, whether VP1 has the cross-protection in Enteroviruses or Coxsackievirus has not been studied in-depth. In this study, the *vp1* gene of Enterovirus 71 (EV71) and Coxsackievirus B3 (CB3) was inserted into the vector pET22b (+) to form the respective expression plasmids pEVP1 or pCVP1, and then transformed into *Escherichia coli* strain BL21 (DE3). The recombinant EVP1 or CVP1 protein was overexpressed successfully and effectively purified to homogeneity. Then, we identified that EVP1 and CVP1 protein could generate effectively specific humoral immunity and cellular immunity in mice, what's more, we determined the cross-protection of VP1 between EV71 and CB3 in a murine model. The results showed that immunization with EVP1 could effectively induce specific IgG and secretory IgA against CVP1 and the sera from EVP1-immunized mice could neutralize CB3 with mean titers 1:440. In contrast, no measurable neutralizing antibodies to EV71 were detected in CVP1-immunized mice. Then, newborn BALB/C mice, whose mother was immunized with EVP1 or CVP1, were administered with different lethal doses of EV71 or CB3. The EVP1 immunized group showed a 90% protective efficacy for a CB3 dosage of 120 LD_50_, but the CVP1 immunized group showed no significantly different protective efficacy against 15 LD_50_ of EV71 compared with the BSA immunized group. Hence, EVP1 is a promising subunit vaccine candidate against Enterovirus 71 and Coxsackievirus B3 caused HFMD.

## Introduction

Hand, foot, and mouthi disease (HFMD) is a childhood illness associated with fever and vesicular eruptions on the hands, feet, and mouth. Numerous large outbreaks of HFMD have occurred in the Asia Pacific region, Europe, and other continents (1–7). HFMD has become a serious public disease in China since 2004 (8–14). Because HFMD has been found to be life-threatening to children and infants, it was listed as a category C notifiable disease in China in May of 2008 (15). The classes of infectious diseases were categorized by the Ministry of Health of the People's Republic of China, with Class A, which includes cholera and the plague, as being the most serious and Class C the least serious.

HFMD is caused by a number of Enteroviruses genus serotypes, such as Enterovirus 71 (EV71), Coxsackievirus A16 (CA16), and Coxsackievirus B3 (CB3). EV71 and CA16 are two major viruses associated with HFMD (16). Children are particularly susceptible to CB3 infection, with aseptic meningitis and myocarditis as the major causes of morbidity and mortality. The mortality rate for infants with myocarditis alone ranges from 30 to 50% and is even higher when other organs are involved (17). However, infection with multiple *Enteroviruses* viruses is also common. Therefore, a preventive strategy to cover more epidemic strains is necessary for clinical protection.

Viral envelope protein 1 (VP1) is one of the major immunogenic capsid proteins of the *Enteroviruses* genus. Several studies have shown the potential of VP1 in both the diagnosis and vaccine development against EV71 and other HFMD viruses (18–23). As a group of closely related RNA viruses, Enteroviruses and Coxsackievirus often exhibit cross immune reactivity and share common antigenic properties (24, 25). A previous study has shown that the specific immunity and protection induced by CA16 can cross-react with EV71 and increase the survival rate of mice after EV71 exposure (26). Thus, whether VP1 also exhibits cross-protection in Enteroviruses and Coxsackievirus still needs to be deeply studied. In the current study, two recombinant VP1 proteins from EV71 and CB3 (EVP1 and CVP1) were produced to determine whether cross-protection was induced between EV71 and CB3.

## Materials and Methods

### Viruses, Bacterial Strains, Plasmids, and Media

Vero cells were maintained in RPMI1640 medium, and the virus of EV71 (GenBank: JQ514785.1) and CB3 (GenBank: M88483.1) were grown in Vero cells. Bacteria were grown in Luria-Bertani (LB) broth or on LB agar (Oxoid LTD, Basingstok, Hampshire, England) supplemented with 100 μg/ml of ampicillin as needed for selection of recombinant plasmid.

### Animal and Ethics Statement

The Beijing Institute of Microbiology and Epidemiology Animal Care and Use Committee approved our study protocol. The animal care and use protocol adhered to the regulations of the Institutional Animal Care and Use Committee (IACUC). BALB/c mice were purchased from the Laboratory Animal Center of the Academy of Military Medical Sciences, Beijing, China. The animals were fed with standard diet and water, maintained under the following conditions: 12 h light/12 h dark controlled lighting, 24–28°C temperature, and 55% relative humidity. All animals were handled under the care and supervision of a veterinarian.

### Construction of Recombinant Plasmids

Viral RNA was extracted with Trizol (Invitrogen, USA) following the manufacturer's instructions. First-strand cDNA was synthesized using reverse transcriptase (Promega) and the evp1 or cvp1 gene was amplified by PCR from the double-stranded cDNA with the respective primers. The primer sequences used were evp1 upstream and downstream primer: (5′-CATATGGGAGATAGGGTGGC-3′, 5′- CTCGAGAAGAGTGGTGATCG-3′), cvp1 upstream and downstream primer: (5′- CATATGGGCCCAGTGGAAGAC-3′, 5′-CTCGAGAAATGCGCCCGTAT-3′). The entire VP1 gene was ligated into pEASY-T1 for sequencing. Then the plasmid was digested by NdeI/XhoI and inserted into the expression vector pET22b (+) to form the expression plasmids pEVP1 or pCVP1. The correct plasmids were transformed into *E. coli* strains BL21 (DE3). DNA sequencing was performed by Sangon Biotech Ceo., Ltd. (Beijing).

### Expression and Purification of Recombinant Protein

*Escherichia coli* BL21 (DE3) containing the expression plasmid was cultured at 37°C in LB broth. Cells were induced at an OD_600_ of 0.6 with β-D-thiogalactopyranoside (IPTG) to a final concentration 1 mM, and grown for additional 6 h at 25°C. The bacteria were then obtained by centrifugation at 5,000 × g for 10 min, and re-suspended in phosphate buffer (pH 7.4 for EVP1, pH 8.0 for CVP1). After bacterial suspension lysis by sonication, cellular debris was separated by centrifugation at 10,000 × g for 20 min at 4°C. Protein was collected and identified by 12% SDS-PAGE to confirm the expression form.

The result of SDS-PAGE showed that the recombinant EVP1 or CVP1 proteins were contained in inclusion bodies of *E. coli*. Then, the separated inclusion body was dissolved in buffer A (8 M urea, 20 mM sodium phosphate, 0.5 M NaCl, 40 mM imidazole, pH 7.4 for EVP1, pH 8.0 for CVP1). The purification was performed with nickel-nitrilotriacetic acid (Ni-NTA) columns according to the manufacturer's instructions (HisTrap, Amersham). After the protein was applied to the column and washed by buffer A, the recombinant protein was eluted by increasing the imidazole concentration to 500 mM. The harvested protein was then dialyzed by serial grades of urea (8, 6, 4, 3, 2, and 1 M) and finally dialyzed into PBS. The protein samples were also confirmed by Western blot with anti-EV71 or CB3 antibody (Santa Cruz Biotechnology, Inc., with concentration of 10 μg/ml).

### Mouse Immunization

Six-weeks-old adult female and male BALB/c mice were divided randomly into four groups, named (1) EVP1, (2) CVP1, (3) EVP1+CVP1, and (4) BSA with 20 mice per group. The animals were boosted three times: at day 0, 14, and 28. We used three doses of VP1 protein to immunize mice. In the low dose group, the first immunization was intraperitoneally (i.p.) injected with a mixture of 12.5 μg EVP1, CVP1, or EVP1+CVP1 (1:1) protein and complete adjuvant, the second and third immunization was injected with a mixture of 25 μg EVP1, CVP1, or EVP1+CVP1 (1:1) protein and incomplete adjuvant. In the medium dose group, the mice were injected with 25, 50, 50 μg protein, and in the high dose group, the mice were injected with 50, 100, 100 μg protein.

### Determination of Anti-EVP1 and Anti-CVP1 ELISA Titers and IgG Subtype Distribution

96-well Costar plates were coated overnight with 100 ng/well of each type of purified VP1 protein at 4°C and blocked with 3% bovine serum albumin (BSA) in PBS for 2 h at room temperature to prevent non-specific binding. Plates were washed with PBST (PBS and 0.5% Tween-20) after which 100 μl serial diluted sera in PBS were added to each well and incubated for 4 h at 4°C. After washing with PBST, the HRP-conjugated secondary antibody was added to wells for another 1 h at 37°C. Finally, after being washed with PBST and developed with TMB, measurements were taken at 450 nm by an absorbance microplate reader.

The method of ELISA (as described above) was performed to determine special antibody subtypes in the mouse serum. Purified EVP1 or CVP1 was used as antigen and serial dilutions of mice sera in BSA were added as different subtypes of antibodies. The detection antibodies were goat anti-mouse IgA-HRP, goat anti-mouse IgG1-HRP, goat anti-mouse IgG2a-HRP, goat anti-mouse IgG2b-HRP, and goat anti-mouse IgG3-HRP.

### Plaque Assay and Determination of Sera Neutralization Titers

A confluent monolayer of Vero cells was prepared in 24-well plates (4 × 10^5^ cells/well). The cells were infected with serial dilutions of EV71 (or CB3), overlaid with 1.5% methylcellulose in DMEM with 2% FBS, and incubated at 37°C for 3 days before the plaques were visualized using crystal violet staining. The 50% tissue culture infective doses (TCID_50_) were determined according to the method described by Zuckerman (27) using the Reed and Muench formula. Neutralizing antibodies titers (NTs) were determined using a micro assay with Vero cells. Briefly, serum samples were collected from immunized mice at day 100 after the first immunization and inactivated for 30 min at 56°C. Fifty microliters of serial serum dilutions (two-fold gradient diluted from 1:10 to 1:5,120) were mixed, respectively, with 50 μl of 100 TCID_50_ EV71 (or CB3) in a 96-well plate, and Vero cell suspensions (final concentration 8 × 10^3^ cells) were added 2 h later. After incubation for 6 days at 37°C, NTs were determined as the highest dilutions of serum that inhibited virus growth.

### Mucosal Immunity in Mice

To identify whether EVP1 or CVP1 protein could induce mice generate mucosal immune, secretory IgA antibody in the mucosal surfaces were measured by ELISA. The intestine samples were collected at 0, 10, 24, 38, 74, and 100 days after the first vaccination, tissues of each immune group were washed with PBS buffer and harvested by centrifugation at 10,000 × g for 30 min. The method of ELISA was performed as described above, the intestinal lavage supernatant was used as antigen, the HRP-conjugated goat anti-mouse IgA was used as the detection antibody.

### Detection of Cytokine Levels in Vaccinated Mice

The measurement of interferon (IFN-γ) was to demonstrate whether EVP1 or CVP1 protein could elicit the cellular immunity. Sera from EVP1, CVP1, EVP1+CVP1, and the BSA-immunized group were collected at days 0, 10, 24, and 38 days after the first immunization. The concentrations of IFN-γ were elevated by ELISA using the Mouse IFN gamma Platinum ELISA kit (Thermo Fisher). The assay sensitivities of cytokines were 15.6–1,000 pg/ml.

### Mouse Model of Protection From Lethal Virus Infection

Adult mice were resistant to EV71 (or CB3) administrations. In this study, the mice, at 1 day of age, were infected with 100 μl of serial dilutions of EV71 or CB3 stock culture by i.p. injection. The mice in the control group were given 100 μl Vero cell lysate and kept in the cages separate from the infected groups. Animals were monitored daily for clinical signs, body weight changes and the occurrence of mortality served as the experimental endpoint. One-day-old models were used to examine the role of maternal antibodies in challenge studies. For maternal immunization, the BALB/C suckling mice possessing passively transferred maternal EV71 or CB3 antibodies were challenged 1 day after birth with the EV71 or CB3 virus. The mice were observed daily for the occurrence of mortality as the experimental endpoint.

### Tissues Collection and Virus Titration

One-day-old suckling BALB/c mice were i.p. injected with EV71 or CB3, the skeletal muscles of mice were removed under sterile condition at days 1, 3, 5, and 7. Tissues were weighed, grinded, and centrifuged, virus titers of skeletal muscle were determined by plaque assays, the method was performed as described above, the plaques were counted and expressed as plaque-forming units per milligram (PFU/mg) tissue.

### Statistical Analysis

The anti-EVP1 or anti-CVP1 antibody titers from the EVP1, CVP1, or EVP1+CVP1 immunized groups were compared to the BSA-immunized group by one-way ANOVA using the program SPSS 13.0. Survival differences in the mice were determined by one-sided Fisher's exact test or the Kaplan–Meier estimator. If the *p*-value was < 0.05, the result was considered significantly different.

### Conformational B-Cell Epitope Prediction of VP1 Protein

Conformational epitopes on the VP1 protein were predicted using DiscoTope 2.0 (28) and EPSVR (29). Cut-off values of epitopes were set at −3.7 (DiscoTope 2.0), and 70 (EPSVR). Consensus sites in the two tools were determined as conformational epitopes.

## Results

### Construction of Plasmids and Expression of EVP1 and CVP1 Proteins

**Figure 1:**
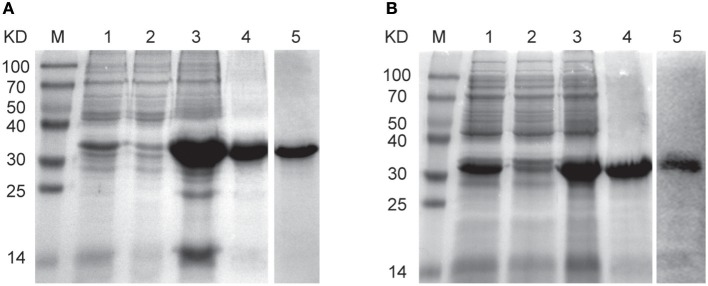
The SDS-PAGE and Western blot results for identifying the expression and purity of EVP1 **(A)** and CVP1 **(B)**. **(A)** M: protein marker; lane 1 is the total bacterial proteins of pET22b (+)-EVP1; lane 2 is the supernatant of bacterial lysates; lane 3 is the precipitation of bacterial lysates; lane 4 is the purified EVP1 protein; lane 5 is Western blot by anti-EV71 antibodies. **(B)** M: protein marker; lane 1 is the total bacterial proteins of pET22b (+)-CVP1; lane 2 is the supernatant of bacterial lysates; lane 3 is the precipitation of bacterial lysates; lane 4 is the purified CVP1 protein; lane 5 is Western blot by anti-CB3 antibodies.

The *evp1* or *cvp1* gene was inserted into the vector pET22b (+) to form the respective expression plasmids pEVP1 or pCVP1. Then the plasmids were transformed into *Escherichia coli* strains BL21 (DE3) for the expression of recombinant EVP1 or CVP1 proteins. The expression of EVP1 or CVP1 was verified in supernatant or precipitation of the bacterial lysates by SDS-PAGE (Figure 1). The results showed that the proteins were present in the precipitation in the form of inclusion bodies. Then the protein renaturation and purification processes were performed to acquire the soluble EVP1 or CVP1 protein with high purity. In addition, the molecular weights matched with the theoretical prediction: 33 kDa for EVP1 (Figure 1A) and 32 kDa for CVP1 (Figure 1B). Moreover, the proteins were, respectively, recognized by anti-EV71 or anti-CB3 antibody in Western blot analysis. The final yield of purified EVP1 or CVP1 was about 20 or 25 mg/L of bacterial culture.

### Immunization With EVP1 Could Effectively Induce Specific IgG and sIgA Against CVP1

**Figure 2:**
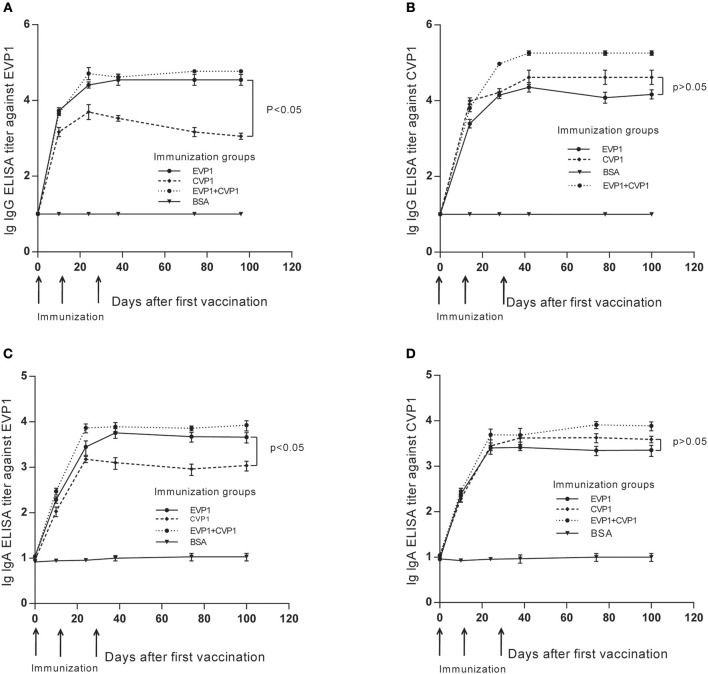
Titers of total IgG and secretory IgA antibodies in the three immunized groups of BALB/C mice changing course detected by EVP1 and CVP1. **(A)** Titers of total IgG in the three immunized groups of BALB/C mice changing course detected by EVP1. **(B)** Titers of total IgG in the three immunized groups of BALB/C mice changing course detected by CVP1. **(C)** Titers of secretory IgA in the three immunized groups of BALB/C mice changing course detected by EVP1. **(D)** Titers of secretory IgA in the three immunized groups of BALB/C mice changing course detected by CVP1. Different symbols represent different immunized groups: EVP1 recombinant protein-immunized group is represented by the black circle; CVP1 recombinant protein-immunized group is represented by the diamond, EVP1+CVP1-immunized group is represented by the star, and the control group immunized with BSA is represented by the inverted triangle. The *p*-value of the statistical analysis in each subtype was marked.

The purified VP1 protein was used to immunize mice by intraperitoneally (i.p.) injection. The animals were boosted 3 times at day 0, 14, and 28 (Figure 2), and the antibody titer changes were examined at day 0, 10, 24, 38, 74, and 100, The results showed that the antibody titers of all immunized groups except the BSA group exhibited a significant increase after vaccinations, peaking at day 38, that is 10 days after the third vaccination (Figure 2). Interestingly, during the observation of the antibody titers against CVP1, the IgG and sIgA titers from the EVP1 and CVP1 immunized groups did not show any significant difference (*p* > 0.05, Figures 2B,D), which indicates that EVP1 immunization could cross-induce specific IgG and sIgA against CVP1, similar to the group immunized only against CVP1. However, for antibody titers against EVP1, the CVP1-immunized group was significantly lower than the EVP1-immunized group (*p* < 0.05, Figures 2A,C), which showed that CVP1 could not effectively induce specific antibodies against EVP1.

**Figure 3:**
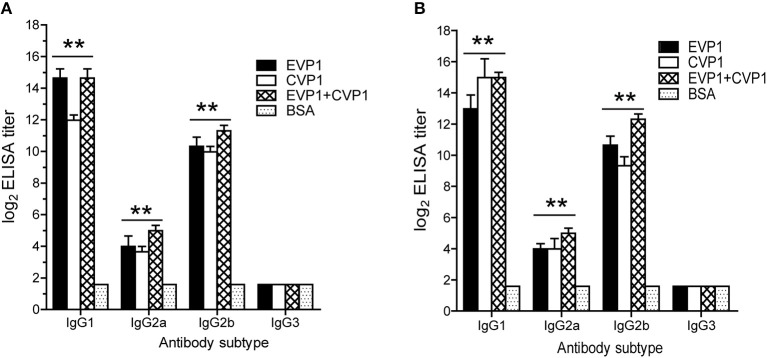
Antibody subtyping. Subtype analysis of the antibodies to EVP1 **(A)** and CVP1 **(B)** in the three immunized groups. Sera from each mouse was obtained at day 100 after the first immunization and tested by ELISA using purified recombinant EVP1 **(A)** and purified recombinant CVP1 **(B)** as antigen. **p < 0.01 vs. BSA group in each subtype.

Then, the serum IgG subtypes caused by each antigen were further delineated, which was based on the sera from each mouse at day 100 after the first immunization. All immunized groups showed a much higher OD value in all IgG subtypes compared with the control group. In addition, the titers of antibodies to either EVP1 or CVP1 had almost the same levels. The immunized groups generated IgG and its subtype IgG1; IgG2b was higher than IgG2a and IgG3 (Figure 3). Although IgA titers were also tested, IgA did not increase significantly compared with the control group.

### VP1 Protein Immune Could Effectively Enhance IFN-γ Production

**Figure 4:**
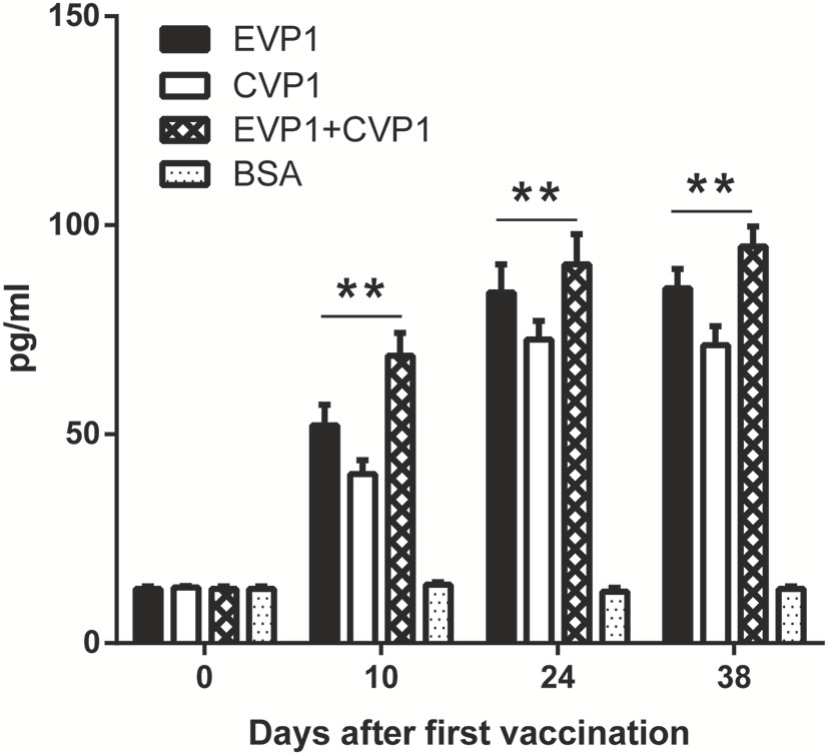
ELISA for measuring IFN-γ of different immunized groups. Sera from EVP1, CVP1, EVP1+CVP1, and the BSA-immunized group were collected at days 0, 10, 24, and 38 after the first immunization. The concentrations of IFN-γ were elevated by ELISA, **p < 0.01 vs. BSA group in each immunization group at day 10, 24, and 38.

To detect the cellular immune responses in vaccinated mice, the concentrations of interferon (IFN-γ) in sera were examined by ELISA at day 0, 10, 24, and 38 (Figure 4). The results showed that the interferon (IFN-γ) levels in the three VP1 protein immunization group increased significantly compared with negative control group (*p* < 0.01, Figure 4) which showed that VP1 protein immune could effectively induce cellular immunity in mice.

### EVP1 Immune Sera Neutralized Both EV71 and CB3 *in vitro*

**Figure 5:**
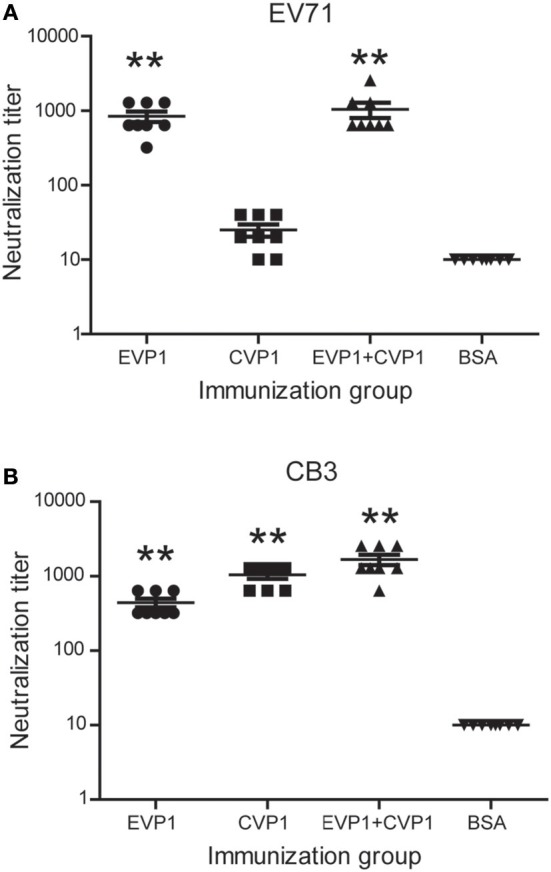
Titers of neutralizing antibodies in three groups of BALB/c mice to EV71 **(A)** and CB3 **(B)**. The serum antibody samples were collected at day 100 after the first immunization. **p < 0.01 vs. BSA group in each immunization group.

The production of antibodies by immunized mice that neutralized the cytotoxicity of the virus to Vero cells was considered as an *in vitro* indicator of the protective immune response. The neutralizing titers of EVP1, CVP1, or EVP1+CVP1 immunized mice are shown in Figure 5. Compared with the control group, all the immune sera generated in adult mice from the EVP1 and CVP1 groups included neutralization antibodies against the corresponding virus with mean neutralization titers reaching 1:840 and 1:1,040, respectively. In addition, the sera of EVP1+CVP1-immunized mice showed neutralizing activity against EV71 or CB3. The neutralization titers of all experimental groups maintained an elevated level for at least 3 months after immunization. Interestingly, the sera from EVP1-immunized mice could neutralize CB3 with mean titers 1:440 (Figure 5B). In contrast, no measurable neutralizing antibodies to EV71 were detected in CVP1-immunized mice (Figure 5A). These data show that the mice immunized with EVP1 produced antibodies that could neutralize both EV71 and CB3, but the CVP1 immune sera could not neutralize EV71.

### Immunization With EVP1 Protected Mice Against EV71 and CB3 Lethal Challenge

**Figure 6:**
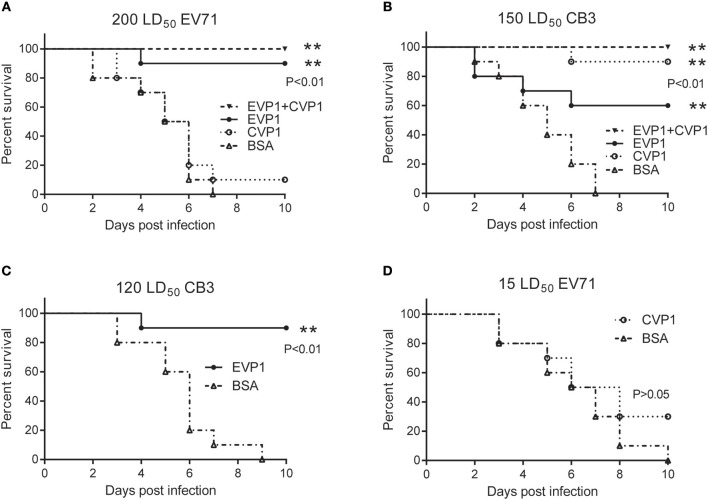
Survival percentage of medium dose immunized groups after exposure to EV71 or CB3 in BALB/C suckling mice. **(A)** Survival of suckling mice born from mothers immunized with (25, 50, 50 μg) EVP1, CVP1, EVP1+CVP1 (1:1), or BSA after exposure to 200 LD_50_ of EV71. *n* = 10 mice per group. **p < 0.01 vs. BSA group in each immunization group. **(B)** Survival of suckling mice born from mothers immunized with (25, 50, 50 μg) EVP1, CVP1, EVP1+CVP1 (1:1), or BSA after exposure to 150 LD_50_ of CB3. **(C)** Survival of suckling mice born from mothers immunized with EVP1 or BSA after exposure to 120 LD_50_ of CB3. **(D)** Survival of suckling mice born from mothers immunized with CVP1 or BSA after exposure to 15 LD_50_ of EV71. *n* = 10 mice per group. The *p*-value of the statistical analysis in each exposure was marked, **p < 0.01.

Hand, foot, and mouth disease (HFMD), which is a common infectious disease worldwide caused by *Enteroviruses*, mostly occurs in infants and young children. Thus, 1-day old mice have often been used to study the protection against HFMD relative viruses, such as enterovirus 71 (23, 26). In this study, the immunized female BALB/C mice were bred at day 7 after the third immunization and produced suckling mice after 4 weeks. Then the newborn BALB/C mice were administered with EV71 or CB3 virus. The survival rates were different in the 4 immunized groups receiving the same doses of virus (Figure 6). At the EV71 virus dosage of 200 LD_50_ (Figure 6A), the EVP1-immunized group showed a protective efficacy of 90%; the EVP1+CVP1-immunized group showed a protective efficacy of 100%, but the CVP1-immunized group only showed a 10% protective efficacy. At the CB3 virus dosage of 150 LD_50_ (Figure 6B), the CVP1-immunized group showed a protective efficacy of 90%; the EVP1+CVP1-immunized group showed a protective efficacy of 100%, and the EVP1-immunized group showed a 60% protective efficacy. To further detect the cross-protection of the VP1 protein, newborn BALB/C mice were administered with lower dosages of EV71 or CB3 (Figures 6C,D). The EVP1-immunized group showed a 90% protective efficacy for a CB3 dosage of 120 LD_50_ (Figure 6C, *p* < 0.01 compared with BSA- immunized group). However, the CVP1-immunized group showed no significantly different protective efficacy against 15 LD_50_ of EV71 compared with the BSA-immunized group (Figure 6D, *p* > 0.05). Similar results were obtained in high and low doses immune group (Figure S1).

**Figure 7:**
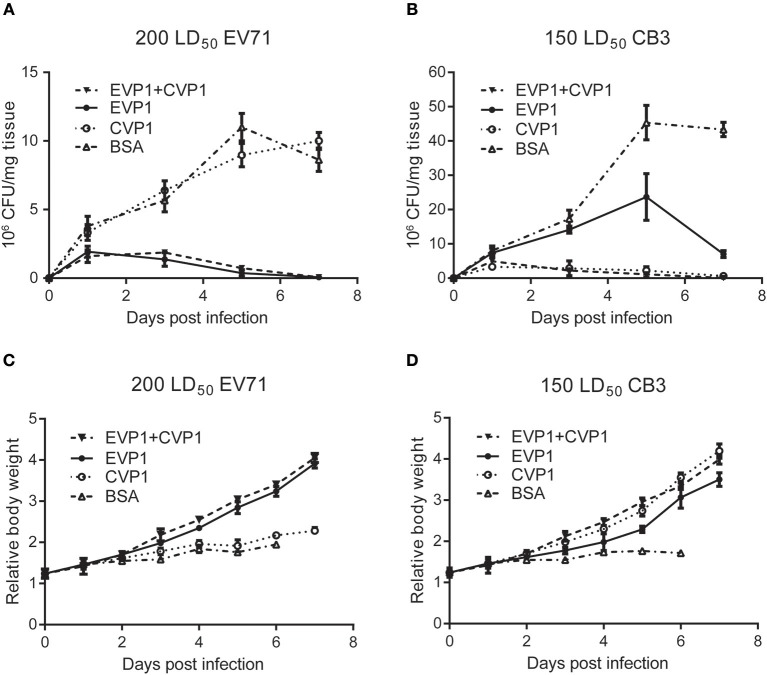
Changes of body weight and virus titers in skeletal muscle of BALB/c suckling mice. **(A)** One-day-old suckling BALB/c mice from mothers immunized with (25, 50, 50 μg) EVP1, CVP1, EVP1+CVP1 (1:1), or BSA were i.p. injected with 200 LD50 EV71. The body weight changes were monitored every day. **(B)** One-day-old suckling BALB/c mice from mothers immunized with (25, 50, 50 μg) EVP1, CVP1, EVP1+CVP1 (1:1), or BSA were i.p. injected with 150 LD50 CB3. The body weight changes were monitored every day. **(C)** One-day-old suckling BALB/c mice from mothers immunized with (25, 50, 50 μg) EVP1, CVP1, EVP1+CVP1 (1:1), or BSA were i.p. injected with 200 LD50 EV71. Skeletal muscles of suckling mice were removed and virus titers were detected at days 1, 3, 5, and 7. **(D)** One-day-old suckling BALB/c mice from mothers immunized with (25, 50, 50 μg) EVP1, CVP1, EVP1+CVP1 (1:1), or BSA were i.p. injected with 150 LD50 CB3. Skeletal muscles of suckling mice were removed and virus titers were detected at days 1, 3, 5, and 7.

Next, we examined the virus titers in skeletal muscle (Figures 7A,B) of the new born mice at different time points post inoculation, virus titers were measured by standard plaque formation assays. At the EV71 virus dosage of 200 LD_50_, virus titers tended to decline after day 1 in EVP1 and EVP1+CVP1 immunized groups, while in the CVP1 and BSA immunized group, virus titers showed an upward tendency to day 5, The virus titers of CVP1-immunized group showed no significantly different with the ones of BSA-immunized group (Figure 7A). While the new born mice were infected with 150 LD_50_ CB3 virus, virus titers tended to decrease after day 1 in CVP1 and EVP1+CVP1 immunized groups. Whereas, virus loads gradually increased from day 1 to day 5, but were significantly different in EVP1 and BSA immunized group. We also examined the body weight changes (Figures 7C,D) of the new born mice daily. The results showed that the loss of total body weight was detected starting at 3 days post infection (dpi) in CVP1 and BSA immunized group at the EV71 virus dosage of 200 LD_50_. However, at the CB3 virus dosage of 150 LD_50_, no significantly different body weight change was observed in EVP1 immunized group compared with CVP1 and EVP1+CVP1 immunized group. All the results indicated that EVP1 immunized mice could produce effective protection against both EV71 and CB3 in the new born mice.

## Discussion

The potential technological advantages of recombinant vaccines over conventional whole virus vaccines have led researchers to find suitable antigen proteins. A previous paper showed that the VP1 proteins of EV71 protected suckling mice against high doses of EV71 (23). In this study, we found that VP1 from CB3 has high neutralization titers and provides protection against CB3 at high dosages. A similar effect was observed with the EVP1-immunized group against EV71. Thus, VP1 could be used as a candidate antigen for Enterovirus 71 and Coxsackievirus B3 vaccine research.

Co-infection with multiple enteroviruses is very common in HFMD. Thus, the cross-protection among Enterovirus and Coxsackievirus has been explored by researchers. In this study, the immunization of two VP1s from EV71 and CB3 were used to determine the cross-protection. The results showed that immunization with EVP1 could effectively induce specific neutralizing antibodies to CB3 and protect newborn mice against lethal doses of CB3. In contrast, the CVP1-immunized groups failed to induce neutralizing antibodies and provide protection against EV71 exposure. To investigate the possible reason underlying the cross-reactivity of EVP1 to CB3 virus, but not CVP1 to EV71 virus, EVP1 and CVP1 protein sequences were aligned and used to predict conformational epitope (Figure S2). One hundred and five consensus sites were identified in 265 aligning sites (identifies: 40%). Two VP1 proteins were predicted using DiscoTope 2.0 and EPSVR, consensus sites in the two tools were determined as conformational epitopes. Twenty-six epitope amino acid positions were found in EVP1 protein sequence which were consistent with previous results (30, 31), and only three sites were located in the homologous region between EVP1 and CVP1. In contrast, among the 62 predicted epitope amino acid positions discovered in CVP1 protein sequence, 17 sites were placed at the homologous region. Combined with the results of mice immune protection experiments, we inferred that the 17 epitopes presented by EVP1 were sufficient to protect mice against lethal doses of CB3 infection, while the only 3 epitopes provided by CVP1 were not sufficient to defend against EV71 virus infection. However, whether these mechanisms actually operate in EV71 and CB3 immunity needs further investigation.

In a previous paper, Wu et al. (26) investigated the use of EV71, CA16, and CB3 inactivated viruses to study the cross-protection in different *Enteroviruses*. A similar result showed that immunization with CB3 failed to provide protection against EV71, although whether EV71 could provide protection against CB3 was not discussed. Their study also showed that immunization with CA16 immune serum increased the survival rate of mice upon EV71 exposure. The amino acid sequence homology for EV71 VP1 and CA16 VP1 was 70.00–72.70% (32); however, the VP1s of EV71 and CB3 strains used in this study shared only 40% homology. The different homology possibly has an important function in the cross-reaction of viruses of different types. Similar studies were also done by using the clinical samples from infected patients. Lin et al. (33) evaluated the cross-reactivity of neutralizing antibodies in series sera of patients infected with EV71 and CA16. The results of neutralization tests showed that 18.9% of CA16-infected patients and 11.1% of EV71-infected patients present high cross-neutralization antibodies against each other. Huang et al. (34) showed that there was no evidence of cross-protection of Enterovirus 71 antibodies against other enteroviruses in kindergarten children in Taipei city. However, due to the absence of circulating EV71 and coxsackievirus A16 in their research period, the relation between seroprevalence and cross-protection of EV71 antibodies was undetermined. Based on the results of those studies, we infer that there is cross-protection in several HFMD relative viruses. Nonetheless, the cross-protection between different Enteroviruses and Coxsackieviruses are very complicated, and it is difficult to explain every result with the existing data. Thus, our finding could be a lead for further investigations of cross-protection by expanding the population of viruses.

In conclusion, the findings suggest that the VP1 protein of EV71 cannot only provide mice protection against challenges from EV71 but also against challenges from CB3. Thus, it might be used as a candidate vaccine to control EV71 and CB3 co-infection.

## Ethics Statement

This study was carried out in accordance with the recommendations of regulations of the Institutional Animal Care and Use Committee (IACUC). The protocol was approved by The Beijing Institute of Microbiology and Epidemiology Animal Care and Use Committee.

## Author Contributions

TL and HW designed the research study. H-LF, F-HC, XL, N-ZN, ZL, NN, and D-YL performed the experiment and data analysis. TL, XL, H-LF, and F-HC wrote the manuscript.

### Conflict of Interest

The authors declare that the research was conducted in the absence of any commercial or financial relationships that could be construed as a potential conflict of interest.

## Supplementary Material

Figure S1 Survival percentage of high (A,B) and low (C,D) dose immunized groups after exposure to EV71 or CB3 in BALB/C suckling mice. Survival of suckling mice born from mothers immunized with (50, 100, 100 μg) EVP1, CVP1, EVP1+CVP1, or BSA after exposure to 200 LD_50_ of EV71. *n* = 10 mice per group. ***p* < 0.01 vs. BSA group in each immunization group. (B) Survival of suckling mice born from mothers immunized with (50, 100, 100 μg) EVP1, CVP1, EVP1+CVP1, or BSA after exposure to 150 LD_50_ of CB3. (C) Survival of suckling mice born from mothers immunized with (12.5, 25, 25 μg) EVP1 or BSA after exposure to 200 LD_50_ of EV71. (D) Survival of suckling mice born from mothers immunized with (12.5, 25, 25 μg) CVP1 or BSA after exposure to 150 LD_50_ of CB3. *n* = 10 mice per group. ***p* < 0.01 vs. BSA group in each immunization group.

Figure S2 Sequence alignment and epitope analysis of EVP1 and CVP1. Amino acid sequence of EVP1 and CVP1 were aligned using online Blastp program, 105/265 consensus amino acids were highlighted in red. VP1 amino acid were also used to predicted conformational B-cell epitopes, 26 and 62 aa positions were identified as epitopes in EVP1 and CVP1, and 3 and 17 aa position were located in consensus sites, the amino acid and the corresponding epitope position were labeled, respectively (top half 1–4: EVP1, bottom half 6–9: CVP1).
