# Supplementary figures and images for: VP1 of Enterovirus 71 Protects Mice Against Enterovirus 71 and Coxsackievirus B3 in Lethal Challenge Experiment
Source: Front Immunol. 2019 Nov 8;10:2564. doi: 10.3389/fimmu.2019.02564 (PMC6856078; doi:10.3389/fimmu.2019.02564)

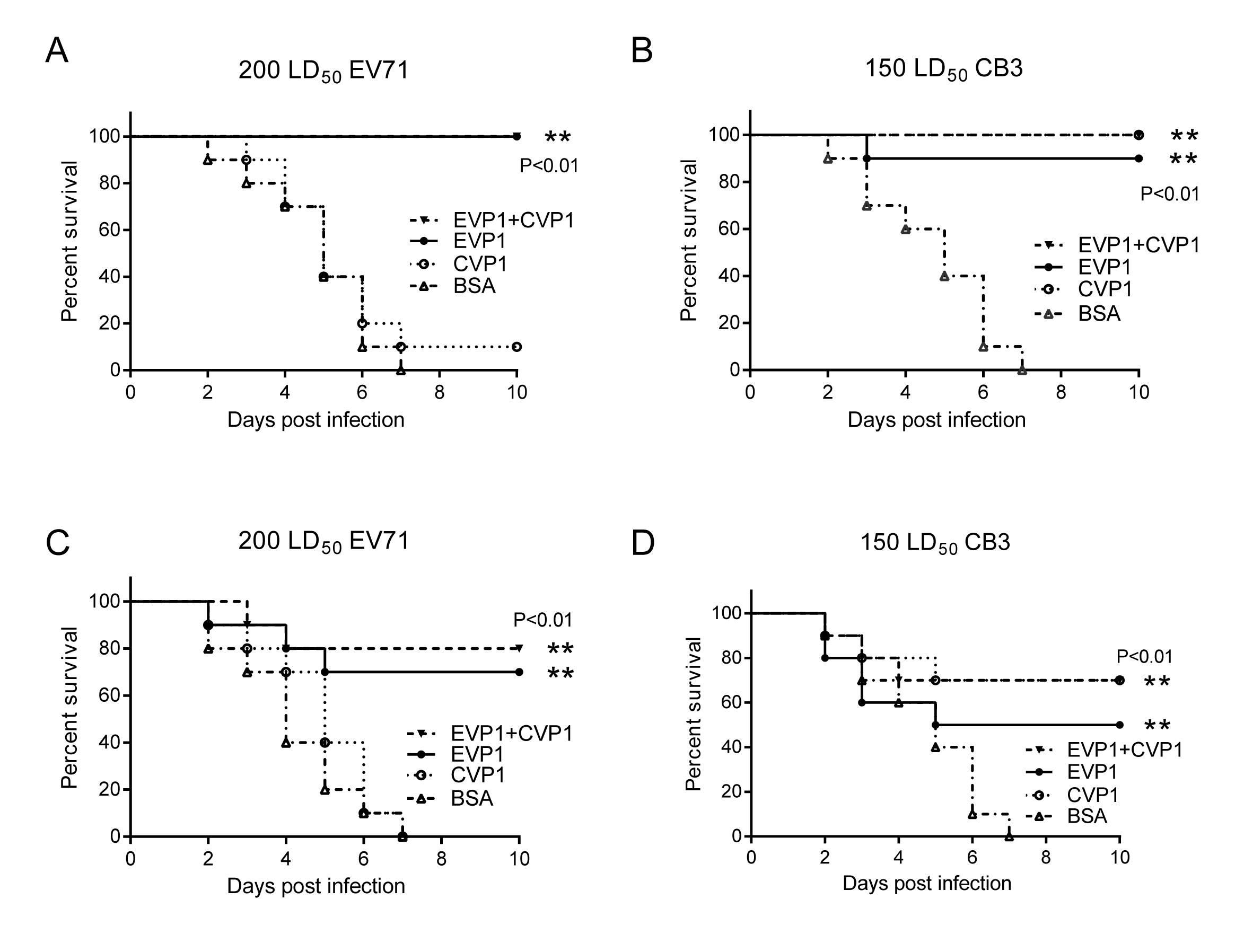

Supplement: Figure S1 — Survival percentage of high (A,B) and low (C,D) dose immunized groups after exposure to EV71 or CB3 in BALB/C suckling mice. Survival of suckling mice born from mothers immunized with (50, 100, 100 μg) EVP1, CVP1, EVP1+CVP1, or BSA after exposure to 200 LD50 of EV71. n = 10 mice per group. **p < 0.01 vs. BSA group in each immunization group. (B) Survival of suckling mice born from mothers immunized with (50, 100, 100 μg) EVP1, CVP1, EVP1+CVP1, or BSA after exposure to 150 LD50 of CB3. (C) Survival of suckling mice born from mothers immunized with (12.5, 25, 25 μg) EVP1 or BSA after exposure to 200 LD50 of EV71. (D) Survival of suckling mice born from mothers immunized with (12.5, 25, 25 μg) CVP1 or BSA after exposure to 150 LD50 of CB3. n = 10 mice per group. **p < 0.01 vs. BSA group in each immunization group. [file Image_1.TIF]

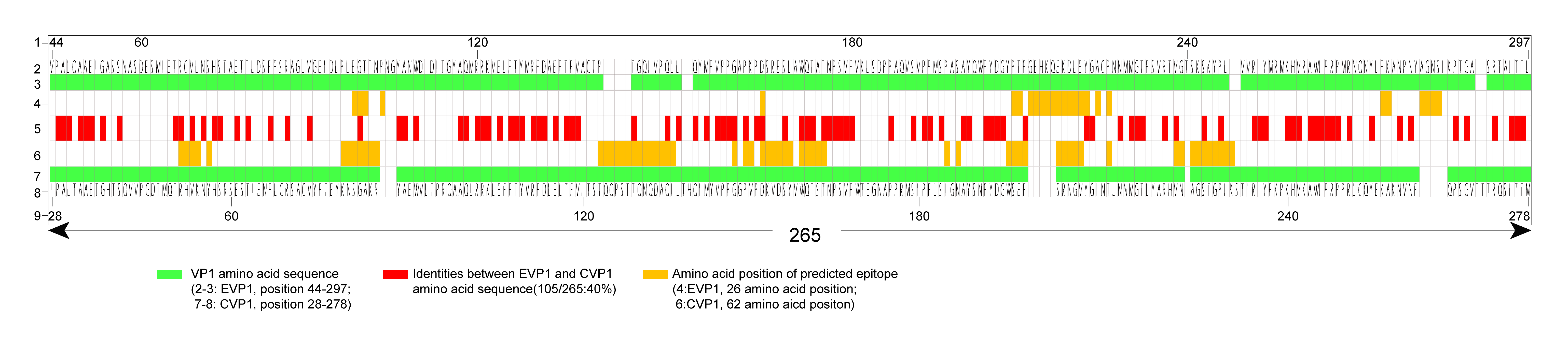

Supplement: Figure S2 — Sequence alignment and epitope analysis of EVP1 and CVP1. Amino acid sequence of EVP1 and CVP1 were aligned using online Blastp program, 105/265 consensus amino acids were highlighted in red. VP1 amino acid were also used to predicted conformational B-cell epitopes, 26 and 62 aa positions were identified as epitopes in EVP1 and CVP1, and 3 and 17 aa position were located in consensus sites, the amino acid and the corresponding epitope position were labeled, respectively (top half 1–4: EVP1, bottom half 6–9: CVP1). [file Image_2.TIF]
